# Medical Expenditures by Body Mass Index Among Privately Insured US Adults Aged 18 to 64 Years, 2022

**DOI:** 10.1001/jamanetworkopen.2025.55436

**Published:** 2026-01-26

**Authors:** Shaonan Wang, Lyudmyla Kompaniyets, Brook Belay, Samantha Lange Pierce, Alyson B. Goodman

**Affiliations:** 1 Division of Nutrition, Physical Activity, and Obesity, Centers for Disease Control and Prevention, Atlanta, Georgia

## Abstract

This cross-sectional study estimates body mass index–associated medical expenditures among privately insured US adults in 2022.

## Introduction

Maintaining a healthy body mass index (BMI; 18.5 to <25.0, calculated as weight in kilograms divided by height in meters squared) is linked to lower medical expenditures and reduced risk of chronic disease.^1^ Yet, from 2015 to 2018, only 26% of US adults had a BMI in the healthy range.^2^ BMI-associated medical costs have not been updated since 2016, and few studies have examined the association of underweight with medical expenditures.^3,4^ This study provides updated estimates of BMI-associated medical expenditures among privately insured US adults in 2022.

## Methods

We linked IQVIA’s PharMetrics Plus claims and ambulatory electronic medical records data to obtain a sample of privately insured US patients aged 18 to 64 years with 1 or more BMI measurements and 11 or more months of continuous enrollment in 2022 (eMethods and eFigure in Supplement 1). The study used deidentified data and institutional review board approval was not required in accordance with 45 CFR §46. The analysis followed the Strengthening the Reporting of Observational Studies in Epidemiology (STROBE) guidelines.

Fractional polynomial regression estimated associations between medical expenditures and continuous BMI, adjusting for sex, age, US Census region, and confounding conditions (eTable in Supplement 1). Generalized linear models were applied to the same outcome with BMI category (reference: healthy weight) and the same covariates. Additional analyses examined differences by sex, age, and setting. A 2-sided *P* < .05 denoted significance. Analyses were conducted using R version 4.5.0 (R Project for Statistical Computing) from March to November 2024.

## Results

Among 704 864 persons, 393 235 (55.8%) were female, and the median (IQR) age was 49 (37-57) years. A total of 217 762 adults (30.9%) had overweight and 314 157 (44.6%) had obesity (including 26 405 adults [3.7%] with BMI ≥45), with 165 587 adults (23.5%) having healthy weight and 7358 (1.0%) having underweight.

Medical expenditures were $1242 higher for individuals with underweight, $317 higher for overweight, and $1776 higher for obesity compared with healthy weight ($8009) (Figure). Excess expenditures were nonuniform within the obesity category, reaching $3388 for BMIs of 45 or higher. In relative terms, expenditures were 16% higher than healthy weight for individuals with underweight (expenditure ratio, 1.16; 95% CI, 1.11-1.20) and 22% higher for obesity (expenditure ratio, 1.22; 95% CI, 1.21-1.23).

**Figure.**
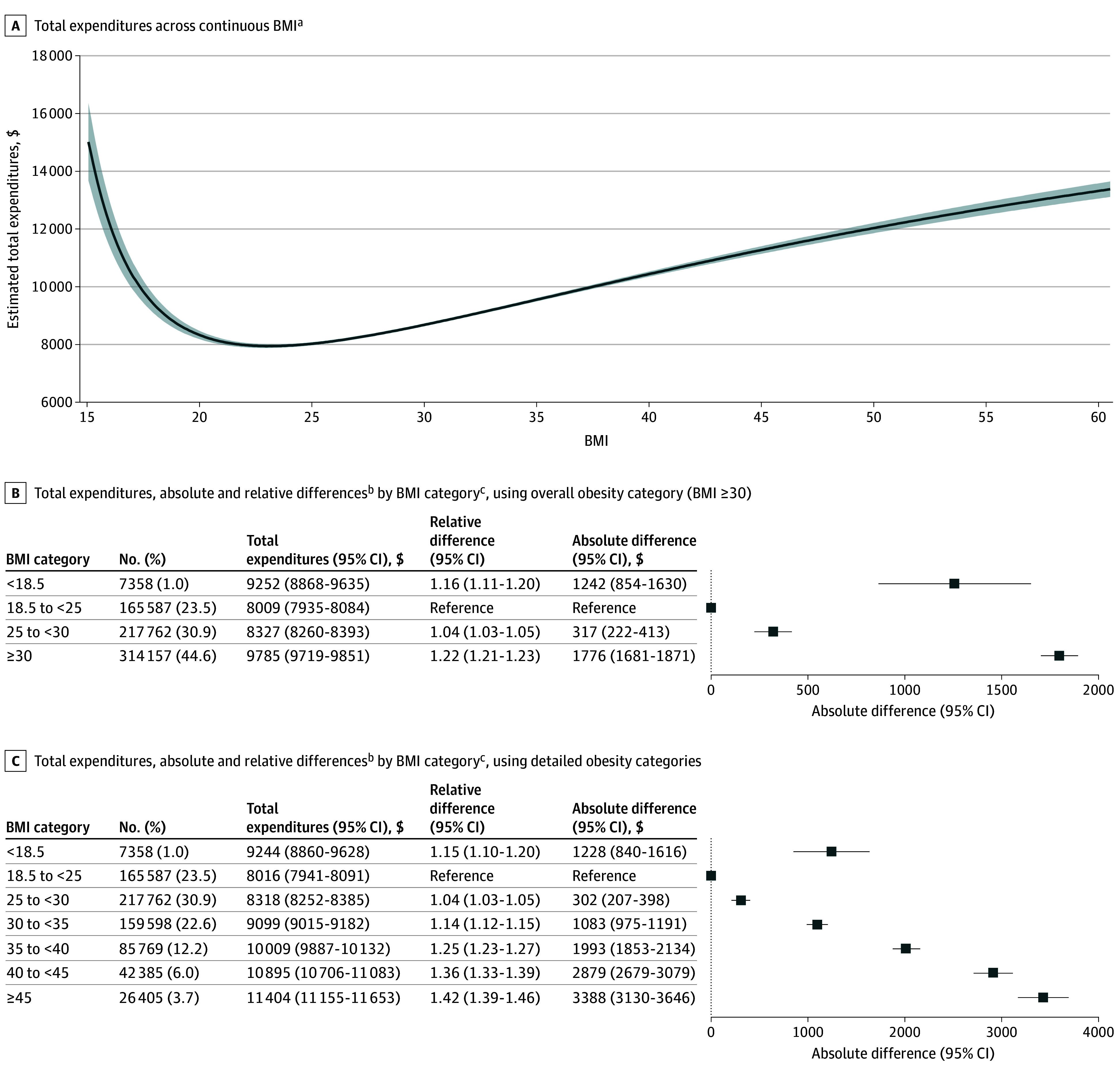
Estimated Absolute and Relative Differences in Expenditures by Body Mass Index Among Privately Insured US Adults Aged 18 to 64 Years, 2022 BMI indicates body mass index (calculated as weight in kilograms divided by height in meters squared). ^a^Estimated expenditures are obtained by fractional polynomial regression adjusting for sex, age, US census region, and confounding conditions. Solid line shows the estimated mean total expenditures across BMI; shaded area indicates 95% CIs. ^b^Estimates come from a generalized linear model (GLM), with total expenditures per person as the outcome variable and BMI category as the covariate of interest, adjusting for sex, age, US census region, and confounding conditions. Absolute differences were calculated as differences in estimated expenditures between each BMI category and the healthy weight category. Relative differences were calculated as ratios of estimated expenditures with the healthy weight category as the reference. ^c^BMI categories were defined as follows: underweight (<18.5), healthy weight (18.5 to <25), overweight (25 to <30), class 1 obesity (30 to <35), class 2 obesity (35 to <40), and 2 categories representing class 3 obesity (40 to <45 and ≥45).

Older age was associated with higher absolute (but not relative) differences in BMI-related expenditures. For individuals with a BMI of 45 or higher vs healthy weight, expenditures were $1843 higher or 35% higher (expenditure ratio, 1.35; 95% CI, 1.22-1.49) at ages 18 to 24 years, and $4146 higher or 42% higher (expenditure ratio, 1.42; 95% CI, 1.36-1.49) at ages 55 to 64 years. Overweight- and obesity-associated excess expenditures were greater for female ($3604) than male ($2995) adults at BMI ≥45 (Table).

**Table.** Absolute Difference and Relative Difference in Total Expenditures by Age, Sex, and Setting Across BMI Categories Among Privately Insured US Adults Aged 18 to 64 Years, 2022

Characteristic	Underweight (BMI <18.5)	Overweight (BMI 25 to <30)	Obesity class			
			1 (BMI 30 to <35)	2 (BMI 35 to <40)	3 (BMI 40 to <45)	3 (BMI ≥45)
**Absolute difference in total expenditures (95% CI), 2022 $ (reference: healthy weight [BMI 18.5 to <25])**						
Age, y^a^						
18-24	377 (−13 to 768)	135 (−49 to 318)	119 (−121 to 359)	272 (−43 to 587)	394 (−45 to 832)	1843 (1152 to 2534)
25-34	772 (0 to 1543)	55 (−134 to 245)	336 (118 to 553)	841 (563 to 1120)	1571 (1185 to 1956)	2130 (1643 to 2617)
35-44	1288 (86 to 2490)	297 (109 to 486)	948 (734 to 1162)	1814 (1541 to 2088)	2650 (2276 to 3023)	2910 (2454 to 3367)
45-54	3389 (1441 to 5337)	286 (77 to 494)	1316 (1087 to 1545)	2089 (1808 to 2370)	3260 (2866 to 3654)	3545 (3044 to 4046)
55-64	2398 (897 to 3899)	469 (255 to 682)	1491 (1254 to 1728)	2860 (2551 to 3169)	3615 (3169 to 4060)	4146 (3520 to 4772)
Sex^a^						
Male	2894 (1190 to 4597)	−51 (−224 to 122)	691 (504 to 877)	1448 (1215 to 1682)	2254 (1920 to 2589)	2995 (2492 to 3498)
Female	1553 (998 to 2107)	582 (454 to 710)	1406 (1258 to 1553)	2447 (2256 to 2639)	3287 (3018 to 3557)	3604 (3275 to 3933)
Expenditure type^b^						
Inpatient	504 (318 to 689)	15 (−26 to 56)	113 (68 to 158)	299 (243 to 355)	451 (375 to 527)	701 (600 to 801)
Pharmacy	435 (202 to 667)	57 (4 to 110)	312 (251 to 373)	632 (551 to 713)	934 (815 to 1052)	1141 (985 to 1296)
Outpatient	349 (140 to 558)	211 (155 to 266)	611 (549 to 673)	980 (901 to 1059)	1304 (1196 to 1412)	1353 (1218 to 1487)
**Relative difference in total expenditures, expenditure ratio (95% CI) (reference: healthy weight [BMI 18.5 to <25])** ^c^						
Age, y						
18-24	1.07 (1.00 to 1.15)	1.03 (0.99 to 1.06)	1.02 (0.98 to 1.07)	1.05 (0.99 to 1.11)	1.08 (0.99 to 1.16)	1.35 (1.22 to 1.49)
25-34	1.13 (1.00 to 1.27)	1.01 (0.98 to 1.04)	1.06 (1.02 to 1.10)	1.15 (1.10 to 1.20)	1.27 (1.20 to 1.34)	1.37 (1.28 to 1.46)
35-44	1.19 (1.01 to 1.37)	1.04 (1.02 to 1.07)	1.14 (1.11 to 1.18)	1.27 (1.23 to 1.32)	1.40 (1.34 to 1.46)	1.43 (1.36 to 1.51)
45-54	1.41 (1.17 to 1.65)	1.03 (1.01 to 1.06)	1.16 (1.13 to 1.19)	1.25 (1.22 to 1.29)	1.39 (1.34 to 1.45)	1.43 (1.37 to 1.49)
55-64	1.25 (1.09 to 1.40)	1.05 (1.03 to 1.07)	1.15 (1.13 to 1.18)	1.29 (1.26 to 1.33)	1.37 (1.32 to 1.42)	1.42 (1.36 to 1.49)
Sex						
Male	1.35 (1.14 to 1.56)	0.99 (0.97 to 1.02)	1.08 (1.06 to 1.11)	1.18 (1.15 to 1.21)	1.27 (1.23 to 1.32)	1.36 (1.30 to 1.43)
Female	1.20 (1.13 to 1.27)	1.07 (1.06 to 1.09)	1.18 (1.16 to 1.20)	1.31 (1.29 to 1.34)	1.42 (1.38 to 1.46)	1.46 (1.42 to 1.51)
Expenditure type						
Inpatient	1.57 (1.35 to 1.78)	1.02 (0.97 to 1.06)	1.13 (1.07 to 1.18)	1.34 (1.27 to 1.41)	1.51 (1.41 to 1.60)	1.79 (1.66 to 1.91)
Pharmacy	1.23 (1.11 to 1.36)	1.03 (1.00 to 1.06)	1.17 (1.13 to 1.20)	1.34 (1.29 to 1.38)	1.49 (1.43 to 1.56)	1.60 (1.52 to 1.69)
Outpatient	1.07 (1.03 to 1.11)	1.04 (1.03 to 1.05)	1.12 (1.11 to 1.13)	1.19 (1.18 to 1.21)	1.26 (1.23 to 1.28)	1.27 (1.24 to 1.29)

Abbreviation: BMI, body mass index (calculated as weight in kilograms divided by height in meters squared).

^a^ Absolute differences (ie, expenditure differences compared with healthy weight) were estimated using generalized linear models (GLM), adjusting for sex, age, US census region, confounding conditions, and a three-way interaction among sex, age, and BMI category.

^b^ Absolute differences were estimated using 2-part models with type-specific expenditures as the outcome, adjusting for sex, age, US Census region, and confounding conditions.

^c^ Relative difference (ie, expenditure ratios compared with healthy weight) is calculated by dividing the estimated expenditure of a BMI category by the estimated expenditure of healthy weight, given a specific age group or sex group.

Among individuals with underweight, the inpatient setting had the highest excess expenditures ($504) compared with outpatient and pharmacy care. Among those with overweight and obesity, the outpatient setting had the highest excess expenditures, reaching $1353 at BMIs of 45 or higher (Table).

## Discussion

In the sample of more than 700 000 privately insured US adults, medical expenditures were higher for individuals with BMIs outside the healthy weight range, reflecting a J-shaped association. Unlike prior studies that found no significant underweight-related costs, we observed excess expenditures, which even exceeded those for class 1 obesity. The acute nature of conditions associated with underweight, such as malnutrition or vascular dysregulation^5^ or underreporting of eating disorders, may explain greater inpatient than outpatient or pharmacy excess expenditures associated with underweight, whereas obesity was associated with higher outpatient costs, consistent with its chronic nature.

The sample reflected health care-seeking adults rather than the general population, which may explain lower obesity-related excess cost estimates than in national survey-based studies.^3,4^ Underreporting of some conditions, such as eating disorders, may have biased underweight-related expenditure estimates.

BMI-related excess expenditures reflect the broader health burden of low and high BMI. Supporting individuals in achieving or maintaining a healthy weight through accessible, evidence-based strategies (eg, lifestyle programs promoting healthy diet and physical activity) could reduce long-term BMI-associated risks and expenditures.

## Notes

Supplement 1. eMethods. eTable. Confounding Conditions Included in Adjusted Models Analyzing the Association Between Body Mass Index and Medical Expenditures Among US Adults Aged 18 to 64 Years in 2022 eFigure. Flowchart of Sample Selection Process for Privately Insured US Adults With Body Mass Index Data in 2022 eReferences

Supplement 2. Data Sharing Statement
