# Supplementary material for: Medical Expenditures by Body Mass Index Among Privately Insured US Adults Aged 18 to 64 Years, 2022
Source: JAMA Netw Open. 2026 Jan 26;9(1):e2555436. doi: 10.1001/jamanetworkopen.2025.55436 (PMC12836126; doi:10.1001/jamanetworkopen.2025.55436)
Supplement: Supplement 1. — eMethods. eTable. Confounding Conditions Included in Adjusted Models Analyzing the Association Between Body Mass Index and Medical Expenditures Among US Adults Aged 18 to 64 Years in 2022 eFigure. Flowchart of Sample Selection Process for Privately Insured US Adults With Body Mass Index Data in 2022 eReferences [file jamanetwopen-e2555436-s001.pdf]

Supplemental Online Content

Wang S, Kompaniyets L, Belay B, Pierce SL, Goodman AB. Medical expenditures by body mass index among privately insured US adults aged 18 to 64 years. *JAMA Netw Open*. 2026;9(1):e2555436. doi:10.1001/jamanetworkopen.2025.55436

eMethods.

eTable. Confounding Conditions Included in Adjusted Models Analyzing the Association Between Body Mass Index and Medical Expenditures Among US Adults Aged 18 to 64 Years in 2022

eFigure. Flowchart of Sample Selection Process for Privately Insured US Adults With Body Mass Index Data in 2022

eReferences

This supplemental material has been provided by the authors to give readers additional information about their work.

© 2026 Wang S et al. *JAMA Network Open*.

eMethods.

Data Source

The IQVIA PharMetrics Plus is a database of adjudicated medical and pharmacy claims for more than 210 million unique privately insured individuals since 2006.[1] It contains inpatient and outpatient diagnoses and procedures, prescription records, associated expenditures, and detailed enrollment histories.[1,2] The IQVIA Ambulatory Electronic Medical Record (AEMR) database contains patient and clinical records for more than 82 million US patients, collected from >100 000 physicians affiliated with >800 ambulatory practices and physician networks.[3] We used the native version of PharMetrics Plus for patient-level insurance and medical expenditure information in 2022 and the Observational Medical Outcomes Partnership Common Data Model version of AEMR for patient demographics and measured height and weight information.

Data Cleaning and Sample Selection

In 2022, we identified approximately 39 million height and weight values for >8 million adults from AEMR (eFigure in Supplement). These data were cleaned longitudinally using the growthcleanr CRAN package that identifies erroneous values.[4,5] Then, BMI was calculated using each person's median height. Heights, weights, and BMIs were excluded if they were substantially higher or lower than expected, defined as height <44 inches (112 cm) or >90 inches (229 cm), weight <25 kg (55 lb) or >454 kg (1000 lb), and BMI <12 or >110. If a patient had multiple BMIs in 2022, one was randomly selected. Next, we merged AEMR and PharMetrics Plus samples and included only 852 739 unique persons with a BMI and 11 months of continuous enrollment in a private insurance plan in 2022.

© 2026 Wang S et al. *JAMA Network Open*.

We excluded (1) pregnant adults, (2) patients aged <18, and (3) adults aged ≥65 because they are eligible for Medicare, a public insurance program, resulting in 714 049 patients in our sample (eFigure in Supplement). Then 9185 (1.3%) persons were excluded if their medical expenditures in 2022 were more than 3 standard deviations ($35 351) above the mean ($11 486) to reduce the influence of potential data errors and implausible outliers.

Confounding Health Conditions

Certain health conditions could simultaneously affect BMI and health care expenditures.[6] For example, hypothyroidism is associated with decreased thermogenesis, metabolic rate, and weight gain.[7-9] Omitting such confounding conditions may bias the expenditure estimates associated with BMI. Therefore, we controlled for these confounding conditions (eTable in Supplement).

These conditions were identified through literature review[10-30] and expert consensus (B.B and A.B.G.). Then we applied the Chronic Conditions Data Warehouse[31] definitions and the Clinical Classifications Software Refined[32] to map these conditions to ICD, Tenth Revision, Clinical Modification (ICD-10-CM) codes. Additionally, some conditions were identified using definitions from previous studies.[11,12,19,26,30] We defined the presence of a condition using the presence of 2 outpatient codes or 1 inpatient code in the Pharmetrics Plus data.

Measures

The study's primary outcome was individual's annual total medical expenditures (the sum of insurance expenditures and patient out-of-pocket [OOP] expenditures). The secondary analysis focused on examining BMI-associated expenditures separately for inpatient, outpatient, and pharmacy categories.

© 2026 Wang S et al. *JAMA Network Open*.

Key independent variables included different specifications of BMI used in separate models: BMI categories or continuous BMI. Covariates included sex (male or female), age (18-24, 25-34, 35-44, 45-54, 55-64 years), confounding conditions (20 binary indicators; eTable), and US Census region (Northeast, Midwest, South, or West). BMI categories were defined as follows: underweight (<18.5), healthy weight (18.5-24.9), overweight (25-29.9), class 1 obesity (30-34.9), class 2 obesity (35-39.9), class 3 obesity (≥40).[33] Within class 3 obesity, we further separated patients into 2 subcategories: 40-44.9 and ≥45, because a sizeable number (3.8%) of patients had a BMI ≥45.

Statistical Analysis

We used generalized linear models (GLM) with gamma distribution and log-link function[34] to estimate total expenditures, accounting for potential heteroscedasticity and high skewness in the data.

$$E(y_i|X_i) = \exp\,(\beta_0 + b_1 BMI_i + \beta_2 Age_i + \beta_3 Sex_i + \beta_4 Region_i + \beta_5 Condition_i + \varepsilon_i)$$

where is $E(y_i|X_i)$ is the expected expenditure for individual i.

We also included a full three-way interaction of BMI category, age, and sex when estimating the expenditures by age and sex. Fractional polynomial regression was applied to model expenditures across continuous BMI.[35] We ran the two-part model for inpatient, pharmacy and outpatient expenditures due to the presence of certain proportions of zero expenditures.[36] To account for zero expenditures, the first part estimated the probability of having positive expenditures using logistic regression. To address the skewed distribution of positive expenditures, the second part was estimated by GLM with a gamma distribution and log-link function, with the dependent variable being positive expenditures. The resulting coefficients

were used to calculate the excess medical expenditures compared with the healthy weight category.

We used the Analytic Research Accelerator to build the cohort and Analytic Dataset Tools to export the data. We conducted the modified Park test and the Box-Cox transformation test to verify the assumptions of our model.[34] All analyses were conducted using R.

The study used deidentified data and institutional review board approval was not required. The analysis adhered to the Strengthening the Reporting of Observational Studies in Epidemiology (STROBE) guidelines.[37]

© 2026 Wang S et al. *JAMA Network Open*.

eTable. Confounding Conditions Included in Adjusted Models Analyzing the Association Between Body Mass Index and Medical Expenditures Among US Adults Aged 18 to 64 Years in 2022[a]

Confounding condition	Definition source			Reference
	CCW	CCSR	Single study	
Hypothyroidism	Y			(10)
Hyperthyroidism			Y	(11)(12)
Pituitary disorders		Y		(13)
Tuberculosis				(14)
Intestinal infection		Y		(15)
HIV infection		Y		(16)(17)
Immunity disorders		Y		(18)
Vitamin B12 deficiency anemia			Y	(19)
Nutritional deficiencies		Y		(20)
Traumatic brain injury; concussion, initial encounter		Y		(21)(22)(23)
Traumatic brain injury; concussion, subsequent encounter		Y		(21)(22)(23)
Injury, sequela		Y		(21)(22)(23)
Epilepsy; convulsions		Y		(24)(25)
Irritable bowel syndrome			Y	(26)
Cancer, breast	Y			(27)(28)
Cancer, lung	Y			(29)
Cancer, bone		Y		(30)
Sarcoma		Y		(30)
Cancer, urologic (kidney, renal pelvis, and ureter)	Y			(30)
Technology dependence			Y	(30)

Abbreviations: CCW, Chronic Conditions Data Warehouse; CCSR, Clinical Classifications Software Refined.

[a] This table presents the confounding conditions and their definition sources that we used to map the International Classification of Diseases (ICD)-10-CM Diagnostic Codes. Specifically, CCW definitions were applied for selected chronic conditions, while CCSR categories were used to group diagnosis codes into clinically meaningful categories for regression.

© 2026 Wang S et al. *JAMA Network Open*.

eFigure. Flowchart of Sample Selection Process for Privately Insured US Adults With Body Mass Index Data in 2022

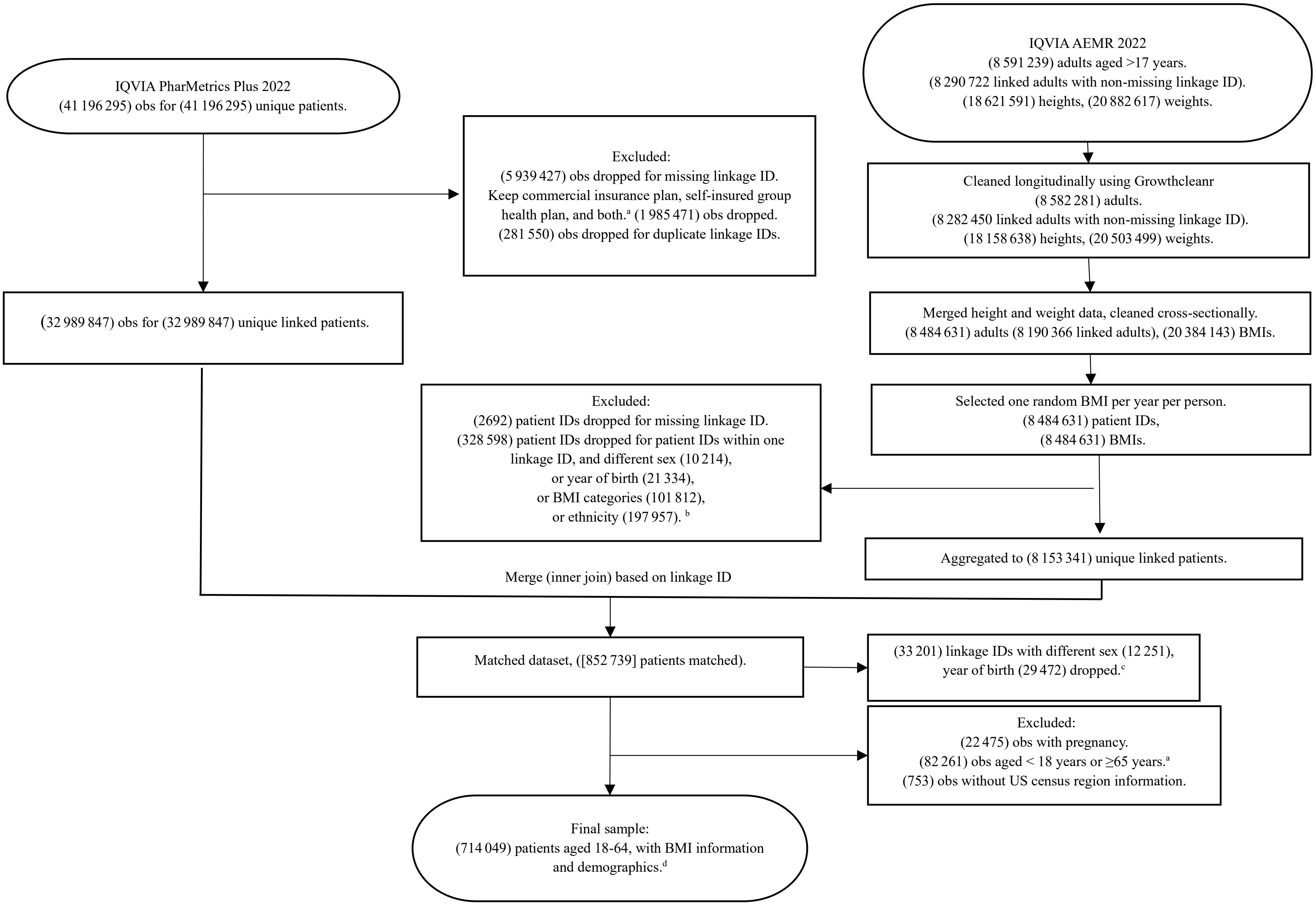

© 2026 Wang S et al. *JAMA Network Open*.

[a] We retained individuals covered under commercial insurance, self-insured group health plans, or both, while excluding other plan types. Some individuals were also covered by Medicare, Medicaid, or other insurance types; these were excluded to make our results comparable. We only focus on privately insured adults aged 18 to 64 Years.

[b] Two identifiers were used: patient ID (unique record for one person) and patient linkage ID (groups multiple patient IDs for the same individual using fuzzy matching). Height, weight, and BMI were cleaned at the patient ID level.

[c] We merged two datasets using patient linkage ID. After merging, records with different age or sex were excluded as potential mismatches.

[d] The analytic sample included 704 864 adults, after excluding 9185 high-expenditure outliers (>3 SD above the mean).

© 2026 Wang S et al. *JAMA Network Open*.

eReferences

[1] IQVIA PharMetrics® Plus Enhanced with Mortality Data. Access date: May 29, 2024. https://www.iqvia.com/-/media/iqvia/pdfs/us/fact-sheet/2023/iqvia-pharmetrics-plus-mortality-fact-sheet-2023.pdf.

[2] Divino V, Ramasamy A, Anupindi VR, et al. Complication-specific direct medical costs by body mass index for 13 obesity-related complications: a retrospective database study. *J Manag Care Spec Pharm*. 2021;27(2):210-222. doi: 10.18553/jmcp.2020.20272

[3] IQVIA Ambulatory EMR-US. Access date: May 29, 2024. https://www.iqvia.com/-/media/iqvia/pdfs/library/fact-sheets/iqvia-ambulatory-emr-us-only.pdf.

[4] Growthcleanr. Access date: May 29, 2024. https://github.com/carriedaymont/growthcleanr.

[5] Daymont D, Ross ME, Russell Localio A, Fiks AG, Wasserman RC, Grundmeier RW. Automated identification of implausible values in growth data from pediatric electronic health records. *J Am Med Inform Assoc*. 2017;24(6):1080-1087. doi: 10.1093/jamia/ocab250.

[6] Kamble PS, Hayden J, Collins J, et al. Association of obesity with healthcare resource utilization and costs in a commercial population. *Curr Med Res Opin*. 2018;34(7):1335-1343. doi: 10.1080/03007995.2018.1464435.

[7] Biondi B. Thyroid and obesity: an intriguing relationship. *J Clin Endocrinol Metab*. 2010;95(8):3614-3617. doi: 10.1210/jc.2010-1245.

[8] Hoogwerf BJ, Nuttall FQ. Long-term weight regulation in treated hyperthyroid and hypothyroid subjects. *Am J Med*. 1984;76:963–970. doi: 10.1016/0002-9343(84)90842-8.

[9] Asvold BO, Bjøro T, Vatten LJ. Association of serum TSH with high body mass differs between smokers and never-smokers. *J Clin Endocrinol Metab*. 2009;94:5023–5027. doi: 10.1210/jc.2009-1180.

© 2026 Wang S et al. *JAMA Network Open*.

[10] Biondi B. Thyroid and obesity: an intriguing relationship. J Clin Endocrinol Metab. 2010; 95(8), 3614-3617. doi: https://doi.org/10.1210/jc.2010-1245.

[11] Dale J, Daykin J, Holder R, Sheppard MC, Franklyn JA. Weight gain following treatment of hyperthyroidism. Clin Endocrinol. 2001; 55(2), 233-239. doi: https://doi.org/10.1046/j.1365-2265.2001.01329.x.

[12] Huynh CN, Pearce JV, Kang L, Celi FS/ Weight gain after thyroidectomy: A systematic review and meta-analysis. Int J Clin Endocrinol Metab. 2021; 106(1), 282-291. doi: https://doi.org/10.1210/clinem/dgaa754.

[13] Dichtel LE, Yuen KC, Bredella MA, Gerweck AV, Russell BM, Riccio AD, et al. Overweight/obese adults with pituitary disorders require lower peak growth hormone cutoff values on glucagon stimulation testing to avoid overdiagnosis of growth hormone deficiency. J Clin Endocrinol Metab. 2014; 99(12), 4712-4719. doi: https://doi.org/10.1210/jc.2014-2830.

[14] Campbell IA, Bah-Sow O. Pulmonary tuberculosis: diagnosis and treatment. Bmj. 2006; 332(7551), 1194-1197. doi: https://doi.org/10.1136/bmj.332.7551.1194.

[15] O'keefe SJD. Nutrition and gastrointestinal disease. Scandinavian journal of gastroenterology. 1996; 31(sup220), 52-59. doi: https://doi.org/10.3109/00365529609094750.

[16] Lam JO, Leyden WA, Alexeeff S, Lea AN, Hechter RC, Hu H, et al. Changes in Body Mass Index Over Time in People With and Without HIV Infection. Open Forum Infect Dis. 2024; 11(2), ofad611. doi: https://doi.org/10.1093/ofid/ofad611.

[17] Crum-Cianflone N, Roediger MP, Eberly L, Headd M, Marconi V, Ganesan A, et al. Increasing rates of obesity among HIV-infected persons during the HIV epidemic. Plos one, 2010; 5(4), e10106. doi: https://doi.org/10.1371/journal.pone.0010106.

© 2026 Wang S et al. *JAMA Network Open*.

[18] De Heredia FP, Gómez-Martínez S, Marcos A. Obesity, inflammation and the immune system. Proc Nutr Soc. 2012; 71(2), 332-338. doi: https://doi.org/10.1017/S0029665112000092.

[19] Dobrozsi S, Flood VH, Panepinto J, Scott JP, Brandow A. Vitamin B12 deficiency: the great masquerader. Pediatr Blood Cancer, 2014; 61(4), 753-755. doi: https://doi.org/10.1002/pbc.24784.

[20] Gebler L, Charuvastra M, Silver D. Nutritional deficiencies associated with obesity. J Obes Weight Loss Ther, 2015; 5(252), 2. doi: http://dx.doi.org/10.4172/2165-7904.1000252.

[21] Izzy S, Tahir Z, Grashow R, Cote DJ, Jarrah AA, Dhand A, et al. Concussion and risk of chronic medical and behavioral health comorbidities. J Neurotrauma. 2021; 38(13), 1834-1841. doi: https://doi.org/10.1089/neu.2020.7484.

[22] Crenn P, Hamchaoui S, Bourget-Massari A, Hanachi M, Melchior JC, Azouvi P. Changes in weight after traumatic brain injury in adult patients: a longitudinal study. Clin Nutr. 2014; 33(2), 348-353. doi: https://doi.org/10.1016/j.clnu.2013.06.003.

[23] Driver S, Douglas M, Reynolds M, McShan E, Swank C, Dubiel R. A narrative review of biopsychosocial factors which impact overweight and obesity for individuals with acquired brain injury. Brain Inj. 2021; 35(9), 1075-1085. doi: https://doi.org/10.1080/02699052.2021.1953596.

[24] Janousek J, Barber A, Goldman L, Klein P. Obesity in adults with epilepsy. Epilepsy Behav. 2013; 28(3), 391-394. doi: https://doi.org/10.1016/j.yebeh.2013.05.012.

[25] Ben-Menachem E. Weight issues for people with epilepsy—a review. Epilepsia. 2007; 48, 42-45. doi: https://doi.org/10.1111/j.1528-1167.2007.01402.x.

[26] Guo YB, Zhuang KM, Kuang L, Zhan Q, Wang XF, Liu SD. Association between diet and lifestyle habits and irritable bowel syndrome: a case-control study. Gut Liver. 2015; 9(5), 649. doi: https://www.ncbi.nlm.nih.gov/pmc/articles/PMC4562783/.

[27] Kroenke CH, Chen WY, Rosner B, Holmes MD. Weight, weight gain, and survival after breast cancer diagnosis. J Clin Oncol. 2005; 23(7), 1370-1378. doi: https://doi.org/10.1200/JCO.2005.01.079.

[28] Rimer BK, Winter, EP. Weight gain in women diagnosed with breast cancer. J Am Diet Assoc. 1997; 97(5), 519-529. doi: https://doi.org/10.1016/S0002-8223(97)00133-8.

[29] Khalid U, Spiro A, Baldwin C, Sharma B, McGough C, Norman AR, et al. Symptoms and weight loss in patients with gastrointestinal and lung cancer at presentation. Support Care Cancer. 2007; 15, 39-46. doi: https://doi.org/10.1007/s00520-006-0091-0.

[30] Kumar A, Kompaniyets L, Belay B, Pierce SL, Grosse SD, Goodman AB. Body mass index and associated medical expenditures in the US among privately insured individuals aged 2 to 19 years in 2018. JAMA Pediatr. 2023; 177(8), 827-836. doi: https://doi.org/10.1001/jamapediatrics.2023.2012.

[31] Chronic Conditions Data Warehouse. Access date: May 29, 2024. https://www2.ccwdata.org/web/guest/home/.

[32] Chronic Condition categories and the Clinical Classifications Software Refined. Access date: May 29, 2024. https://hcup-us.ahrq.gov/toolssoftware/ccsr/ccs_refined.jsp.

[33] Centers for Disease Control and Prevention. BMI categories for adults. Access date: May 1, 2025. https://www.cdc.gov/bmi/adult-calculator/bmi-categories.html.

[34] Deb P, Norton EC. Modeling health care expenditures and use. *Annu Rev Public Health*. 2018;39:489-505. doi: 10.1146/annurev-publhealth-040617-013517.

[35] Royston P, Altman DG. Regression using fractional polynomials of continuous covariates: parsimonious parametric modelling. *J R Stat Soc Ser C Appl Stat*, 1994;43(3):429-453. doi: 10.2307/2986270.

[36] Belotti F, Deb P, Manning WG, Norton EC. twopm: Two-part models. *Stata J*, 2015;15(1):3-20. doi: 10.1177/1536867X1501500102.

© 2026 Wang S et al. *JAMA Network Open*.

[37] Von Elm E, Altman DG, Egger M, Pocock SJ, Gøtzsche PC, Vandenbroucke JP. The strengthening the reporting of observational studies in epidemiology (STROBE) statement: guidelines for reporting observational studies. *Ann Intern Med*. 2007;147(8):573-577. doi:10.7326/0003-4819-147-8-200710160-00010

© 2026 Wang S et al. *JAMA Network Open*.
