# Supplementary material for: Medical Expenditures by Body Mass Index Among Privately Insured US Adults Aged 18 to 64 Years, 2022
Source: JAMA Netw Open. 2026 Jan 26;9(1):e2555436. doi: 10.1001/jamanetworkopen.2025.55436 (PMC12836126; doi:10.1001/jamanetworkopen.2025.55436)
Supplement: Supplement 2. — Data Sharing Statement [file jamanetwopen-e2555436-s002.pdf]

Data Sharing Statement

Wang. Medical Expenditures by Body Mass Index Among Privately Insured US Adults Aged 18 to 64 Years, 2022. *JAMA Netw Open*. Published January 26, 2026.
doi:10.1001/jamanetworkopen.2025.55436

Data
Data available: No

Additional Information
Explanation for why data not available: The data used in this study were obtained from IQVIA and are not publicly available due to licensing restrictions. Individual-level data and the data dictionary cannot be shared.
